# Using structural equation modelling to jointly estimate maternal and fetal effects on birthweight in the UK Biobank

**DOI:** 10.1093/ije/dyy015

**Published:** 2018-02-13

**Authors:** Nicole M Warrington, Rachel M Freathy, Michael C Neale, David M Evans

**Affiliations:** 1 University of Queensland Diamantina Institute, The University of Queensland, Translational Research Institute, Brisbane, QLD, Australia; 2 Institute of Biomedical and Clinical Science, University of Exeter Medical School, University of Exeter, Royal Devon and Exeter Hospital, Exeter, UK; 3 Medical Research Council Integrative Epidemiology Unit, University of Bristol, Bristol, UK; 4 Virginia Institute for Psychiatric and Behavioral Genetics, Departments of Psychiatry and Human & Molecular Genetics, Virginia Commonwealth University, Richmond, VA, USA; 5 School of Social and Community Medicine, University of Bristol, Bristol, UK

**Keywords:** Structural equation model, maternal effects, fetal effects, birthweight, UK Biobank

## Abstract

**Background:**

To date, 60 genetic variants have been robustly associated with birthweight. It is unclear whether these associations represent the effect of an individual’s own genotype on their birthweight, their mother’s genotype, or both.

**Methods:**

We demonstrate how structural equation modelling (SEM) can be used to estimate both maternal and fetal effects when phenotype information is present for individuals in two generations and genotype information is available on the older individual. We conduct an extensive simulation study to assess the bias, power and type 1 error rates of the SEM and also apply the SEM to birthweight data in the UK Biobank study.

**Results:**

Unlike simple regression models, our approach is unbiased when there is both a maternal and a fetal effect. The method can be used when either the individual’s own phenotype or the phenotype of their offspring is not available, and allows the inclusion of summary statistics from additional cohorts where raw data cannot be shared. We show that the type 1 error rate of the method is appropriate, and that there is substantial statistical power to detect a genetic variant that has a moderate effect on the phenotype and reasonable power to detect whether it is a fetal and/or a maternal effect. We also identify a subset of birthweight-associated single nucleotide polymorphisms (SNPs) that have opposing maternal and fetal effects in the UK Biobank.

**Conclusions:**

Our results show that SEM can be used to estimate parameters that would be difficult to quantify using simple statistical methods alone.

**Key Messages**

- We describe a structural equation model to estimate both maternal and fetal effects when phenotype information is present for individuals in two generations and genotype information is available on the older individual.
- Using simulation, we show that our approach is unbiased when there is both a maternal and fetal effect, unlike simple linear regression models. Additionally, we illustrate that the structural equation model is largely robust to random measurement error and missing data for either the individual’s own phenotype or the phenotype of their offspring.
- We describe how the flexibility of the structural equation modelling framework will allow the inclusion of summary statistics from studies that are unable to share raw data.
- Using the structural equation model to estimate the maternal and fetal effects of known birthweight-associated loci in the UK Biobank, we identify three loci that have primary effects through the maternal genome and six loci that have opposite effects in the maternal and fetal genomes.

## Introduction

Birthweight is a complex trait, and low birthweight is robustly associated with increased risk of a range of cardiometabolic diseases in later life.^1^ It has long been known that birthweight is under the influence of both maternal and fetal genetic sources of variation. Using a large sample consisting of the offspring of twins, Magnus illustrated that more than 50% of the variation in birthweight is caused by fetal genes and less than 20% was caused by maternal genes.^2^ Subsequent studies have reported lower proportions of the variance explained by both fetal and maternal genes, but all have shown that the fetal contribution is larger than the maternal contribution.^3^^,^^4^ Using a method that partitions trait variance into components due to the maternal and fetal genomes,^5^ we reported that common genetic variants in the fetal genome explained approximately 28% of the variation in birthweight, whereas common genetic variants in the maternal genome only explained approximately 8% of the total variance.^6^

We and others have begun to investigate the specific regions of the genome that influence fetal growth, using genome-wide association studies (GWAS). In a recent GWAS meta-analysis combining data from the Early Growth Genetics consortium (EGG; http://egg-consortium.org/) and the UK Biobank,^7^ we identified 60 single nucleotide polymorphisms (SNPs) associated with birthweight at genome-wide levels of significance.^6^ One difficulty we faced in interpreting our results was that it was often not clear whether genetic associations reflected the effect of an individual’s own genotype on their birthweight, an effect of their mother’s genotype on their birthweight (i.e. maternal genotype mediated through the intrauterine effect) or some combination of both. For example, rare mutations in the *GCK* gene, which cause a defect in the sensing of glucose by the pancreas, have radically different associations with birthweight according to their parent of origin. If inherited paternally, birthweight is lower due to reduced glucose sensing and consequent reduced insulin secretion, which results in reduced growth. But if maternally inherited (i.e. present in both mother and fetus), birthweight is close to the population average because the maternal hyperglycaemia compensates for the fetal defect in glucose sensing. In the case that the mother has hyperglycaemia due to a GCK mutation, but the fetus does not inherit the mutation, the birthweight is higher due to normal glucose sensing and thus above-average insulin secretion. This example reflects contrasting effects mediated through the intrauterine environment (i.e. maternal effects) and direct effects of the offspring’s genotype.^8^

In an attempt to resolve this question, in Horikoshi *et al.*^6^ we first performed a simple linear regression of an individual’s self-reported birthweight on their own genotype; and then for the UK Biobank women, we performed a linear regression of the birthweight of their firstborn child on their own genotype. We then compared the maternal and fetal effect sizes to get an idea of whether the locus was operating through the maternal or the individual’s own genotype. However, this approach was suboptimal since it did not consider the correlation between maternal and offspring genotypes, and therefore did not accurately estimate the relative importance of these two potential sources of variation. We also examined the genetic associations with birthweight in cohorts that had genotype information on both mother and offspring. Performing an analysis of offspring birthweight on maternal genotype and conditioning on offspring genotype should yield an unbiased estimate of the mother’s genetic influence on her child’s birthweight, and likewise a regression of offspring birthweight on offspring genotype conditioning on maternal genotype should produce an unbiased estimate of the fetal contribution on birthweight. The difficulty however is that there is a paucity of cohorts in the world that have birthweight data as well as genotype data on both mothers and children, meaning that such an analysis is likely to have low power to resolve maternal and fetal effects.

To better estimate the maternal and fetal genetic contributions to birthweight for each of the 60 genome-wide significant variants reported in Horikoshi *et al.*^6^ we used a structural equation modelling (SEM) approach with birthweight data from the UK Biobank. Our method enables us to model both grand-maternal and offspring genotypes (which were absent in the UK Biobank) as latent factors, and to estimate maternal and fetal effects on birthweight in the same statistical model. To investigate the properties of our approach, we first performed a series of simulations to: (i) quantify any bias in the effect estimates for the maternal and fetal effects; and (ii) estimate power to detect maternal and fetal effects and type 1 error. We also assessed the effect of allele frequency and measurement error in birthweight (which can often be an issue with self-report) on our estimates. We show that our method provides accurate estimates of maternal and fetal effects under a range of different scenarios, and increased power to detect genetic association when maternal and fetal effects operate in opposite directions. We also show how our framework can easily combine summary results data from additional cohorts, including previous large scale GWAS meta-analyses, involving either maternal or offspring phenotypes. Using the UK Biobank data,^7^ we provide strong evidence to suggest that several of the known birthweight-associated SNPs exert effects acting in opposite directions on birthweight through the maternal and fetal genotypes.

## Methods

### Simulations

**Figure 1:**
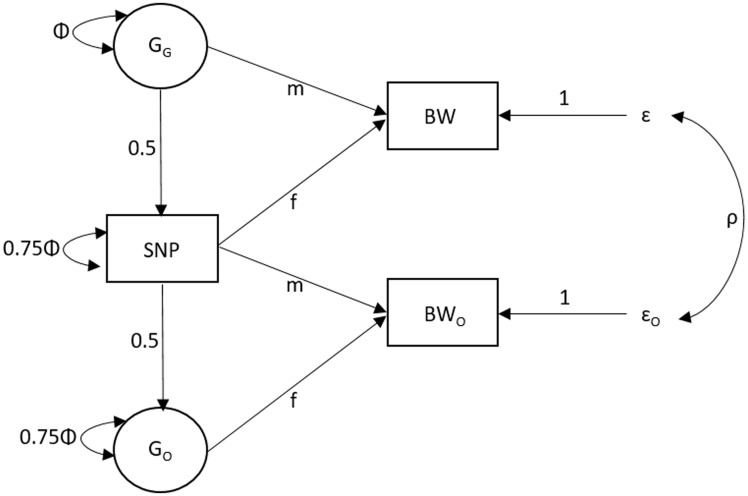
Diagram of the structural equation model (SEM) used for the simulation study and the UK Biobank analysis of birthweight. The three observed variables (in squares) are the birthweight of the individual (BW), the birthweight of their offspring (BW_O_) and the genotype of the individual (SNP). The latent variables (in circles) are the genotypes for the individual’s mother (G_G_) and the genotype of the individual’s first offspring (G_O_). The total variance of the latent genotypes for the individual’s mother (G_G_) and offspring (G_O_) and for the observed SNP variable is set to Φ [i.e. variance(G_G_) = Φ, variance (SNP) = 0.75Φ + 0.25Φ, variance (G_O_) = 0.75Φ + 0.25Φ]. The m and f path coefficients refer to maternal and fetal effects, respectively. The residual error terms for the birthweight of the individual and their offspring are represented by ɛ and ɛ_O_, respectively, and we estimate the variance of both of these terms in the SEM. The covariance between residual genetic and environmental sources of variation is given by *ρ*.

We performed simulations to investigate the bias, power and type one error rate of the SEM for modelling both the individual’s own genetic effect (referred to as the ‘fetal effect’) and maternal genetic effects on birthweight. The model we used for generating the data is illustrated in Figure 1, and the R code used for performing these simulations is provided in the Supplementary material (available as Supplementary data at *IJE* online).

For each scenario, we generated 10 000 replicates of 30 000 maternal-offspring pairs. For each replicate we generated grandparental (on the maternal side) and paternal genotypes at a single locus. Assuming autosomal Mendelian inheritance, additivity and unit variance, latent variables for the genotype of the individual’s mother (i.e. grand-maternal genotype; G_G_), the individual’s own genotype (SNP) and offspring’s genotype (G_O_) were generated. The individual’s own birthweight variable (BW), for each family *i*, was generated using the following equation:

$${BW}_{i}\;=\;\sqrt{V_{M}}\;\times \;G_{G_{i}}+\;\sqrt{V_{O}}\;\times \;{SNP}_{i}+\;\beta_{U}\;\times \;U_{i}+\;\varepsilon_{i}$$

where *V_M_* denotes the variance in birthweight explained by the maternal genotype (‘maternal effect’), *G_G_* is a latent variable indexing the genotype of the individual’s mother, *V_O_* is the variance in birthweight explained by the individual’s own genotype (‘fetal effect’), *SNP* is the genotype of the individual, *U* is a standard normal random variable representing all residual genetic and environmental sources of similarity between mother and offspring, *β_U_* is the total effect of *U* on the individual’s own birthweight and *ɛ* is a random normal variable with mean zero and variance needed to ensure that BW has unit variance asymptotically.

Similarly, offspring birthweight (BW_O_), for each family *i*, was generated using the following equation:

$${BW}_{O_{i}}\;=\;\sqrt{V_{M}}\;\times \;{SNP}_{i}+\;\sqrt{V_{O}}\;\times \;G_{O_{i}}+\;\beta_{UO}\;\times \;U_{i}+\;\varepsilon_{O_{i}}$$

where *G_O_* is a latent variable indexing the offspring genotype, *β_UO_* is the total effect of *U* on offspring birthweight and *ɛ_O_* is a random normal variable with mean zero and variance needed to ensure that BW_O_ has unit variance asymptotically.

In all simulations, the regression of phenotype on residual shared genetic and environmental factors was set to 0.5 (i.e. *β_U_* = *β_UO_* = 0.5). We considered the effects of: allele frequency (p = 0.99, p = 0.90 or p = 0.5); the strength of the fetal genetic effect on birthweight (*V_O_* = 0%, *V_O_* = 0.02% or *V_O_* = 0.04%); and the strength of the maternal genetic effect on birthweight (*V_M_* = 0%, *V_M_* = 0.01% or *V_M_* = 0.02%). We simulated the fetal and maternal genetic effects to have both increasing and decreasing effects on birthweight.

For each simulated dataset we fit a series of models:

- linear models regressing either the individual’s own birthweight or, for the women, the birthweight of their offspring on the SNP (individual’s own genotype), which respectively estimate the fetal and maternal genetic effects on birthweight; this is equivalent to the model typically used in genetic studies of birthweight^6^ and was used for comparison purposes;
- SEM estimating both maternal and fetal effects as illustrated in Figure 1; *P*-values were calculated using Wald tests;
- SEM estimating only the fetal effect; this model was fit to conduct a likelihood ratio test for the maternal effect and the likelihood ratio test *P*-value was compared with the Wald test *P*-value for the fetal effect;
- SEM estimating only the maternal effect, to conduct a likelihood ratio test for the fetal effect; this likelihood ratio test *P*-value was compared with the Wald test *P*-value for the maternal effect;
- SEM with neither fetal nor maternal paths (i.e. both fixed to zero); this model was fit to conduct a likelihood ratio test of the overall SNP effect, and the *P*-value from this test is referred to as the two degrees of freedom (2DF) test *P*-value.

Bias was defined as the mean difference between the estimated SNP effect and the true parameter across the 10 000 simulations, and was calculated for both the maternal and fetal effects. A 95% confidence interval was calculated around the bias to give an indication of the uncertainty in the estimate. Power was defined as the proportion of tests that reached *P* < 0.05 under the alternative hypothesis, and type 1 error rate the proportion of tests that reached *P* < 0.05 under the null hypothesis.

### Additional simulations investigating measurement error

In the UK Biobank, female participants were asked to report the birthweight of their first offspring to the nearest pound. After appropriate data cleaning, this left six discrete birthweight values for the offspring (see below). We therefore conducted a second set of simulations to investigate the effect of this type of measurement error, using the same method as described above but rounding the birthweight of the offspring to the nearest unit.

Given that birthweights of both individuals and their offspring are self-reported in the UK Biobank, we also assessed the potential effect of measurement error on both variables. To do this, we added a normally distributed error component to both simulated birthweight measurements, which is referred to as discrimination or classical measurement error.^9^ For example, an individual’s own birthweight with measurement error was generated as follows:

$${BW}_{i}^{*}\;=\;{BW}_{i}+\;\tau_{i},\;\tau_{i}∼N(0,\delta^{*})$$

where δ* was chosen to produce a specific R^2^ value for the regression of BW* on BW, using the following equation:

$$R^{2}\;=\;\frac{Var(BW)}{Var\left(BW\right)+Var(\tau )}$$

${BW}_{o_{i}}^{*}$ was generated in the same way. We varied the value of *R*^2^ (1.00, 0.75, 0.50, 0.25), where lower values of *R*^2^ represent increasing measurement error. We used a subset of maternal and fetal effect sizes to get an idea of whether the impact of measurement error is influenced by effect size; neither a maternal or fetal effect (*V_O_* = 0% = *V_M_*), large fetal effect and no maternal effect (*V_O_* = 0.04%, *V_M_* = 0%), no fetal effect and a large maternal effect (*V_O_* = 0%, *V_M_* = 0.02%), large fetal and maternal effect (*V_O_* = 0.04%, *V_M_* = 0.02%) and large fetal and maternal effect in opposite directions. All simulations were conducted with an allele frequency of p = 0.5.

### Additional simulations investigating missing data

We also assessed the impact of when individuals did not have both their own and their offspring birthweight available. Supplementary Figure 1 (available as Supplementary data at *IJE* online) illustrates the three components of the SEM used to incorporate individuals with missing data; the first component models individuals with complete data, the second component models genotyped individuals who report their own phenotype but not their offspring’s, and the third component models genotyped mothers who report their offspring phenotype data but not their own. These three components are fit to the three subsets of data and then the likelihoods from each model are combined. Modelling the data in this way avoids list-wise deletion of cases due to missing phenotype information and makes maximum use of the observed data. If data are missing at random then our full information maximum likelihood approach returns asymptotically unbiased parameter estimates, and the most precise estimates that have this property.^10^ We simulated four additional scenarios, all with minor allele frequency of p = 0.5 and a total sample size of 30 000 individuals: (i) 15 000 individuals with both their own and their offspring’s birthweight and 15 000 individuals with their own birthweight only; (ii) 15 000 individuals with both their own and their offspring’s birthweight and 15 000 individuals with their offspring’s birthweight only; (iii) 15 000 individuals with both their own and their offspring’s birthweight, 7500 individuals with their own birthweight only and 7500 individuals with their offspring’s birthweight only; and (iv) 15 000 individuals with their own birthweight only and 15 000 individuals with their offspring’s birthweight only (i.e. no individuals with both birthweight measures, and therefore only the second and third components of Supplementary Figure 1 are fit and the term *ρ* in Figure 1 can not be estimated). Given that we observed very close correspondence between the likelihood ratio and Wald tests, we only conducted Wald tests because these were computationally easier to perform.

### UK Biobank

UK Biobank phenotype data were available on 502 643 individuals, of whom 279 959 reported their own birthweight at either the baseline or follow-up visits. There were 7693 individuals who were part of multiple births and were excluded from the analyses. Of the 9034 individuals who reported their own birthweight at both baseline and follow-up, 401 (4% of individuals with repeat birthweight reports) were excluded because the two values differed by more than 0.5 kg. For those individuals who reported different values between baseline and follow-up (<0.5 kg) we took the baseline measure for the analyses. Finally, we excluded individuals who reported their own birthweight to be <2.5 kg or >4.5 kg [24 138 (9%) individuals with birthweight <2.5 kg and 14 065 (5%) individuals with birthweight >4.5 kg], as these are implausible for live term births before 1970. In total, 233 662 individuals had data on their own birthweight matching our inclusion criteria.

Women in the UK Biobank were also asked to report the birthweight of their first child to the nearest pound. We used the same inclusion criteria as for their own birthweight, leaving 210 405 individuals with birthweight of their first child [51 (0.6%) excluded because the multiple reports of offspring birthweight differed by >0.5 kg; 5838 (3%) excluded with offspring birthweight <2.5 kg; and 473 (0.2%) excluded with offspring birthweight >4.5 kg], 109 205 of whom had also reported their own birthweight.

Genotype data from the May 2015 release were available on a subset of 152 248 individuals. In addition to the quality control metrics performed centrally by the UK Biobank, we excluded individuals who were related. We defined a subset of ‘White European’ ancestry samples using a *K*-means (K = 4) clustering approach based on the first four genetically determined principal components. A subset of 89 296 unrelated individuals with genotype data, a valid birthweight for themselves or their first child and genetically of ‘White European’ ancestry were included in the analysis. Of these, 24 962 were men who only reported their own birthweight. Among the women, 8723 reported only their own birthweight, 24 645 reported only that of their first child and 30 966 reported both. We adjusted both the individual’s own birthweight and the birthweight of their first child for the principal components that were associated with birthweight, adjusted the individual’s own birthweight for sex (sex was not reported for the offspring) and then created z-scores. A subset of 58 autosomal SNPs out of the 60 birthweight-associated SNPs^6^ were extracted from the imputed files provided by UK Biobank and aligned to the birthweight-increasing allele (rs62240962 was not available and rs11096402 is on the X chromosome). We fit the SEM to the data from each of these 58 autosomal SNPs to estimate the maternal and fetal genetic effects on birthweight. To confirm our results, we compared them with those from a conditional linear regression model in a subset of 12 909 individuals with both maternal and offspring genotype data from the EGG consortium, as presented in Horikoshi *et al.*^6^ Specifically, Horikoshi *et al.*^6^ reported: (i) the association between maternal genotype and offspring birthweight after conditioning on offspring genotype (i.e. their estimate of the maternal effect); and (ii) the association between offspring genotype and offspring birthweight after conditioning on maternal genotype (i.e. their estimate of the fetal effect).

## Results

### Bias

**Figure 2:**
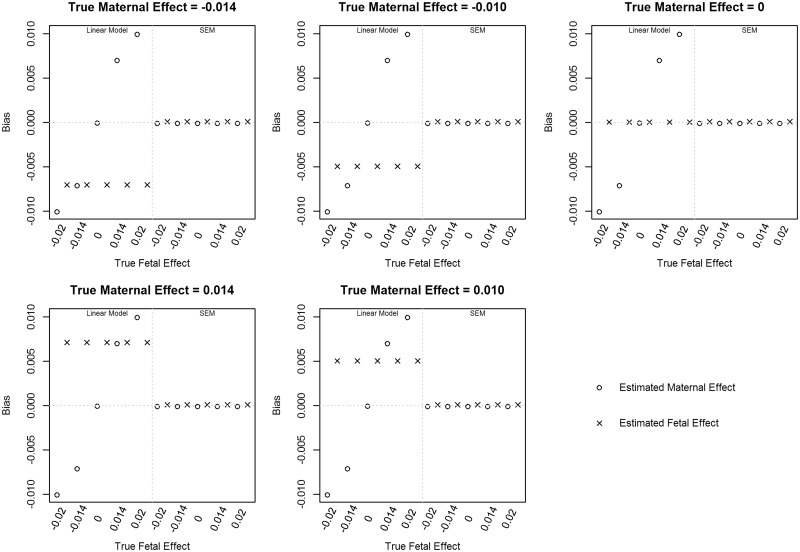
Bias in effect estimates with an allele frequency of 0.5 and varying maternal and fetal effect sizes using two linear models that assess the maternal and fetal effects independently (left panel), or the structural equation model (SEM, right panel) assessing both the maternal and fetal effects simultaneously.

Figure 2 shows the bias calculated from the simulations for the linear model and the SEM in the simulations with allele frequency of 0.5 and all 30 000 individuals whom had complete data for both their own birthweight and the birthweight of their offspring. The fetal effect estimates from the standard linear model are biased wherever there is a maternal effect that is not being modelled. For example, in the scenarios where there is both a fetal and maternal effect, the estimated fetal effect approximately equals the true fetal effect plus half the true maternal effect. In other words, the bias of the estimated fetal effect is approximately half the true maternal effect. In the scenarios where there is no maternal effect, then the fetal effect estimated from the linear model is unbiased. The same pattern of bias occurs for the maternal effect estimates. Conversely, the SEM is unbiased for both the maternal and fetal effects as it simultaneously models both effects. The bias and 95% confidence intervals for all simulation results are presented in Supplementary Table 1 (available as Supplementary data at *IJE* online).

When there was measurement error in either the individual’s own birthweight or the birthweight of their offspring, the estimates of maternal and fetal effects were unbiased (Table 1 for abridged results, and full results in Supplementary Table 2, available as Supplementary data at *IJE* online). However, there was a decrease in the precision of the estimate (i.e. increase in the standard error) as the measurement error increased (Table 1 for abridged results, and full results in Supplementary Table 2). For a small number of scenarios where the birthweight of the offspring was distributed as it is in the UK Biobank, a small bias was introduced from the SEM (Supplementary Table 3, available as Supplementary data at *IJE* online); this bias differed across allele frequency and true effect size for both the maternal and fetal effects, and no clear pattern was observed. The bias was less than 4% of the true value in all scenarios and substantially lower than the bias introduced in the linear models.

**Table 1.** The effect of measurement error in the individual’s own birthweight and the birthweight of their offspring on bias, precision and power in the structural equation model (SEM)

Measurement error		$\sqrt{V_{O}}=0;\sqrt{V_{M}}$**= 0**			$\sqrt{V_{O}}=0;\sqrt{V_{M}}$**=** −**0.014**			$\sqrt{V_{O}}=-0.020;\sqrt{V_{M}}$**= 0**			$\sqrt{V_{O}}=-0.020;\sqrt{V_{M}}$**=** −**0.014**			$\sqrt{V_{O}}=0.020;\sqrt{V_{M}}$**=** −**0.014**		
R^2^ BW	R^2^ BW_O_	Mean estimate	Mean standard error	Power	Mean estimate	Mean standard error	Power	Mean estimate	Mean standard error	Power	Mean estimate	Mean standard error	Power	Mean estimate	Mean standard error	Power
**Estimated fetal effect**																
Measurement error for offspring birthweight only																
1.00	1.00	0.000	0.008	0.048	0.000	0.008	0.048	−0.020	0.008	0.738	−0.020	0.008	0.738	0.020	0.008	0.741
1.00	0.75	0.000	0.008	0.050	0.000	0.008	0.049	−0.020	0.008	0.711	−0.020	0.008	0.712	0.020	0.008	0.702
1.00	0.50	0.000	0.009	0.048	0.000	0.009	0.051	−0.020	0.009	0.655	−0.020	0.009	0.656	0.020	0.009	0.634
1.00	0.25	0.000	0.010	0.048	0.000	0.010	0.048	−0.020	0.010	0.515	−0.020	0.010	0.516	0.020	0.010	0.490
1.00	UKBB^a^	0.000	0.008	0.047	0.000	0.008	0.047	−0.020	0.008	0.733	−0.020	0.008	0.736	0.020	0.008	0.732
Measurement error for individual’s own birthweight only																
1.00	1.00	0.000	0.008	0.048	0.000	0.008	0.048	−0.020	0.008	0.738	−0.020	0.008	0.738	0.020	0.008	0.741
0.75	1.00	0.000	0.009	0.048	0.000	0.009	0.049	−0.020	0.009	0.616	−0.020	0.009	0.618	0.020	0.009	0.612
0.50	1.00	0.000	0.011	0.047	0.000	0.011	0.047	−0.020	0.011	0.448	−0.020	0.011	0.449	0.020	0.011	0.453
0.25	1.00	0.000	0.015	0.046	0.000	0.015	0.046	−0.020	0.015	0.248	−0.020	0.015	0.248	0.020	0.015	0.253
**Estimated maternal effect**																
Measurement error for offspring birthweight only																
1.00	1.00	0.000	0.008	0.052	−0.014	0.008	0.456	0.000	0.008	0.052	−0.014	0.008	0.456	−0.014	0.008	0.457
1.00	0.75	0.000	0.009	0.047	−0.014	0.009	0.350	0.000	0.009	0.047	−0.014	0.009	0.351	−0.014	0.009	0.351
1.00	0.50	0.000	0.011	0.046	−0.014	0.011	0.245	0.000	0.011	0.046	−0.014	0.011	0.246	−0.014	0.011	0.245
1.00	0.25	0.000	0.015	0.047	−0.014	0.015	0.142	0.000	0.015	0.047	−0.014	0.015	0.142	−0.014	0.015	0.142
1.00	UKBB^a^	0.000	0.008	0.052	−0.014	0.008	0.426	0.000	0.008	0.052	−0.014	0.008	0.424	−0.014	0.008	0.433
Measurement error for individual’s own birthweight only																
1.00	1.00	0.000	0.008	0.052	−0.014	0.008	0.456	0.000	0.008	0.052	−0.014	0.008	0.456	−0.014	0.008	0.457
0.75	1.00	0.000	0.008	0.050	−0.014	0.008	0.418	0.000	0.008	0.051	−0.014	0.008	0.418	−0.014	0.008	0.418
0.50	1.00	0.000	0.009	0.051	−0.014	0.009	0.375	0.000	0.009	0.051	−0.014	0.009	0.374	−0.014	0.009	0.375
0.25	1.00	0.000	0.010	0.050	−0.014	0.010	0.283	0.000	0.010	0.051	−0.014	0.010	0.283	−0.014	0.010	0.283

a UKBB indicates offspring birthweight distributed as in the UK Biobank, where only six discrete values are available.

In simulations where either the individual’s own birthweight or that of their offspring was missing, the SEM continued to produce unbiased estimates (Supplementary Table 1). However, in the simulations where all individuals only had their own birthweight or the birthweight of their offspring (i.e. no individuals had both birthweight measures), we detected a small bias in the maternal effect estimate (bias approximately −0.0003, or less than 3% of the true value).

### Power/type 1 error

The power and type 1 error results for all simulations from the SEM and the linear models for the fetal and maternal effects are presented in Supplementary Table 1.

**Figure 3:**
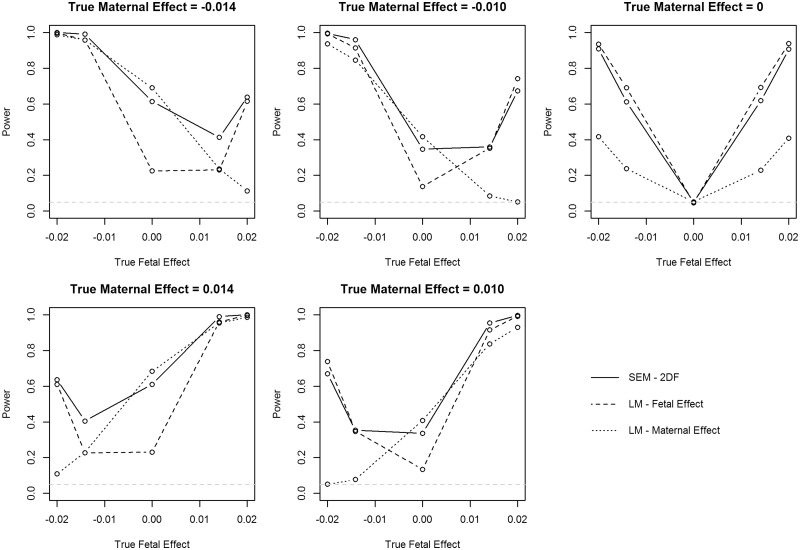
Power of the two degrees of freedom test using the structural equation model (SEM) assessing both the maternal and fetal effects simultaneously, and power of the two linear models (LM) that assess the maternal and fetal effects independently. Note, power from the linear models is artificially inflated due to the bias in the effect estimate, but they are presented here as a comparison with what would be provided from a standard genetic analysis of birthweight. Power is presented for simulations with a minor allele frequency of 0.5.

The linear model has greater power than the Wald test in the SEM when the SNP has either a fetal or maternal effect only (i.e. when the effect estimate is unbiased; Supplementary Table 1). For example, the power is greater for the fetal effect estimated using the linear model over the Wald test from the SEM when the maternal effect is zero. Nevertheless, there is still substantial power to detect an effect using the Wald test in the SEM with α = 0.05, with 74% power to detect a variant that explains 0.04% of the variance, 45% power for one explaining 0.02% of the variance and 25% power for one explaining 0.01% of the variance, in a sample of 30 000 individuals with both their own and their offspring’s birthweight. However, the two degrees of freedom test has very similar power to the linear model when the SNP had either a fetal or maternal effect only, and greater power in most scenarios than testing either maternal or fetal effects individually using the Wald test (Figure 3 and Supplementary Table 1). It is worth nothing that the power estimates from the linear models are artificially inflated due to the bias introduced in the linear models; however, we have included them in the figure as they give an indication of what the power of a standard genetic analysis would be. This indicates that the SEM can detect when a SNP affects birthweight, but it has lower power to detect whether the effect is driven by the mother or the offspring. For example, when the variant explains 0.04% of the variance using the individual’s own genotype and 0.02% of the variance using the mother’s genotype, with α = 0.05, the SEM has 74% power to detect the fetal effect, 45% power to detect the maternal effect and 100% power to detect any effect of the variant using the two degrees of freedom test in a sample of 30 000 individuals with both their own and their offspring’s birthweight. Similarly, with α = 0.05 and 30 000 individuals with complete data, when the variant explains 0.02% of the variance using the individual’s own genotype and 0.01% of the variance using the mother’s genotype, the SEM has 45% power to detect the fetal effect, 25% power to detect the maternal effect and 95% power to detect any effect of the variant using the two degrees of freedom test.

Power for both fetal and maternal effects is reduced when there is measurement error in either the individual’s own birthweight or the birthweight of their offspring, due to the decrease in precision of the estimate (Table 1 and Supplementary Table 2). This decrease in power was the same across the different true effect sizes for the fetal and maternal effects.

**Figure 4:**
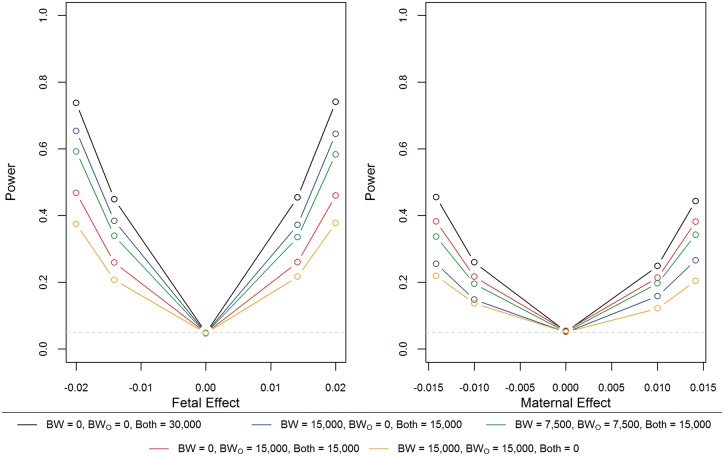
Power from the structural equation model (SEM) with different combinations of individuals reporting their own birthweight (BW) or their offspring’s birthweight (BW_O_). Power for the fetal effect is presented from the simulations where there is no maternal effect; however, similar estimates were obtained when there was a maternal effect (see Supplementary Table 1 for full results). Similarly, for the maternal effect, results are presented from simulations where there is no fetal effect. Power is presented for simulations with a minor allele frequency of 0.5.

Figure 4 shows the power when not all individuals have complete data for both maternal and offspring birthweight. Power is greatest when information is available on both the individual’s own and their offspring’s birthweight; however, there is only a small decrease in power to detect the fetal effect when information on the offspring birthweight is not available in 50% of the individuals, and for power to detect the maternal effect when the individual’s own birthweight is not available in 50% of the individuals. Interestingly, the SEM can still be used to estimate maternal and fetal effects when the sample consists of some individuals only measured on their own birthweight, and others who have only reported the birthweight of their offspring. However, the power to detect either a fetal or a maternal effect is approximately half of that when birthweight data are available on both individuals in the pair.

As expected, the type 1 error from the linear model is inflated in situations where the estimated effect is biased (Supplementary Table 1). However, the type 1 error is well controlled when using the SEM. It remains controlled when birthweight of the offspring is distributed as in the UK Biobank (Supplementary Table 3), when there is measurement error in either the individual’s own birthweight or the birthweight of their offspring (Table 1, Supplementary Table 2) or when data are not available on both the individuals own birthweight and their offspring’s birthweight (Supplementary Table 1).

The difference between *P*-values estimated using the Wald test and the likelihood ratio test in the SEM was negligible (Supplementary Table 1 for mean difference), indicating that the Wald test was adequate.

### Timing

The SEM can be fitted with either the raw data or observed covariance matrices. As seen in Supplementary Figure 2 (available as Supplementary data at *IJE* online), the computational time is approximately 100 times faster using the covariance matrices than the raw data. There is not a substantial difference in computational time between datasets with different amounts of missing data for the phenotype of the individual or their offspring when fitting the model using the raw data, but there is a difference for variants with lower minor allele frequencies which take longer to run than common variants. When using covariance matrices, however, it takes slightly longer to fit the model when data are missing for either the individual or their offspring, because the model fits two or three sub-models simultaneously (i.e. one for each of the complete data subsets: one for individuals with both phenotypes, one for individuals with their own phenotype only and one for individuals with their offspring’s phenotype only). The estimates from the model fit with the raw data are the same as those using with covariance matrices. In comparison with a linear model, the SEM using covariance matrices takes about three times as long to compute with a sample size of 30 000 individuals (Supplementary Figure 2).

### UK Biobank

**Figure 5:**
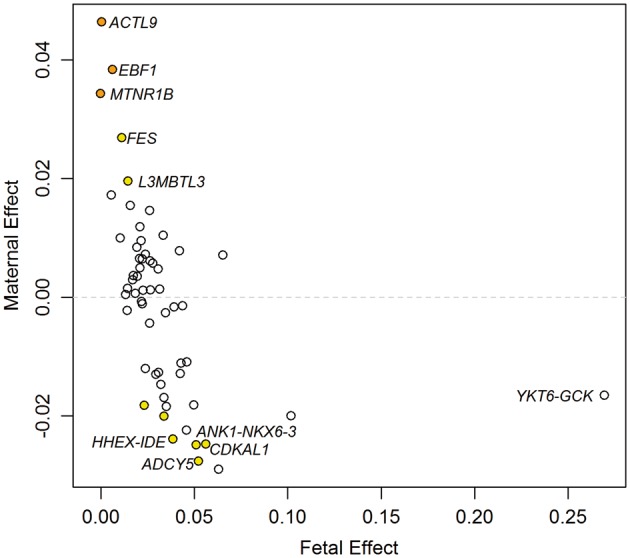
Fetal and maternal effect size estimated using the structural equation model (SEM) for the 58 birthweight-associated SNPs in the UK Biobank. All SNPs are aligned to the birthweight-increasing allele reported in Horikoshi *et al.*^6^ The colour of each dot indicates the maternal genetic association *P*-value for birthweight generated using the Wald test: orange, *P* < 0.001; yellow, 0.001 ≤ *P* < 0.05; white *P* ≥ 0.05. Gene names are provided for those loci with large effects.

Figure 5 presents the results from the SEM for each of the 58 birthweight-associated SNPs in the UK Biobank. It is evident that most of the 58 SNPs only have evidence for a fetal effect, which is unsurprising given how the SNPs were selected. Three SNPs primarily have a maternal effect (*EBF1*, *ACTL9* and *MTNR1B*) and eight SNPs have evidence for both. Perhaps the most interesting SNPs are those where the birthweight-increasing allele identified in Horikoshi *et al.*^6^ has opposite effects on birthweight through the fetal and maternal genotype, half of which are known type 2 diabetes loci (*HHEX-IDE*, *CDKAL1*, *ADCY5* and *ANK1-NXK6-3*).

Supplementary Table 4 and Supplementary Figure 3 (available as Supplementary data at *IJE* online) present the full results from the two linear models (fetal and maternal effects) and the SEM. These results show that for SNPs where the maternal and fetal effects go in opposite directions (for example, the *HHEX-IDE*, *CDKAL1*, *ADCY5* and *ANK1-NKX6-3* loci), the fetal effect estimated in the GWAS^6^ would have been reported to be smaller than its true effect.

To confirm our results, we compared estimates of maternal and fetal effects obtained from the SEM with those from the conditional linear regression model implemented in Horikoshi *et al.*^6^ in the subset of EGG cohorts with both maternal and fetal genotype data (*N* = 12 909 individuals). Supplementary Figure 4 (available as Supplementary data at *IJE* online) displays forest plots for the maternal and fetal effects for each of the 58 birthweight-associated SNPs, using both the SEM in the UK Biobank and conditional linear regression in the EGG cohorts. The confidence intervals surrounding the estimates from the conditional linear regression analyses are larger than those from the SEM, due to the smaller sample size in the former study (12 909 individuals in the conditional regression analysis versus 89 296 individuals in the SEM). Estimates obtained using both procedures were similar for most SNPs. Formally, after Bonferroni correction for the 58 tests, no significant heterogeneity was detected between estimates from the SEM and estimates from the conditional linear regression for either the maternal or the fetal effects (heterogeneity *P* > 0.05/58 = 9 x 1 0^−4^). The largest heterogeneity between the SEM and the conditional regression for the maternal effect was at the *ACTL9* locus (I^2^ = 90.2%, *P* = 0.001), where the conditional linear regression resulted in a (non-significant) negative estimate of the effect of the maternal T allele on offspring birthweight, whereas the SEM resulted in a positive estimate of the effect of the same allele on offspring birthweight. However, the result from the maternal GWAS analysis presented in Horikoshi *et al.*^6^ showed a similar direction of effect as the SEM. It may be that there are differences between EGG and the UK Biobank in how birthweight is measured or analysed, which may be responsible for this discrepancy (for example, many studies in EGG correct birthweight for gestational age whereas this is not done in UK Biobank). Further investigation of this locus needs to be undertaken before any strong conclusions can be drawn.

## Discussion

In this article, we have presented a method for estimating and testing maternal and fetal effects. The approach uses data from mother-offspring pairs for whom genotype data are available for the mothers only and phenotype data are available on both individuals. Our method is (asymptotically) unbiased when both maternal and fetal effects exist, which improves on the traditional linear model which estimates each effect separately while assuming the other to be absent. The approach is flexible and can be used when either the individual’s own phenotype or the phenotype of their offspring is not available. The ability to incorporate studies with only an individual’s own or their offspring’s phenotype, in combination with mother/offspring pairs with complete data, will transform many aspects of perinatal research, as it provides a large increase in statistical power to disentangle maternal and fetal effects which have been difficult to resolve until now.

Data from males are included in the SEM in two ways. First, genotyped males who report their own phenotype (birthweight), but not their offspring’s phenotype, are modelled in the top half of Figure 1. For example, these males contributed directly to estimation of the fetal effect of genotype on birthweight (see the coefficient labelled ‘f’ that is on the path from their own SNP to their own BW as illustrated in the top half of Figure 1) and also indirectly to estimation of the maternal effect on birthweight, since their observed genotype (SNP) is correlated with their mother’s unmeasured latent genotype at the same locus (G_G_) (see the coefficient ‘m’ that is on the path from SNP to G_G_ to BW in the top half of Figure 1). Second, male data contribute to estimation in this SEM when the offspring of a UK Biobank female with genotype data is male and she reports his phenotype (i.e. birthweight of offspring BW_O_). For example, these offspring males contribute directly to the estimation of the maternal effect on birthweight (see the coefficient ‘m’ that is on the path from SNP to BW_O_ in the lower half of Figure 1) and indirectly to the estimate of the fetal effect on birthweight, since the male’s latent genotype (G_O_) is correlated with his mother’s observed genotype at the same locus (SNP) (see the coefficient ‘f’ that is on the path from SNP to offspring genotype G_O_ to offspring birthweight BW_O_ in the lower half of Figure 1). It is important to note that the inclusion of males in this way is not equivalent to estimating a paternal effect. To estimate paternal effects, one would need information on males’ own genotype, their own phenotype and their offspring’s phenotype. Our SEM purely involves resolving maternal and fetal effects.

To illustrate the method, we used birthweight because there is clear evidence that both maternal and fetal effects exist.^2–4^^,^^11^ However, the method could be useful for many other phenotypes, especially pregnancy outcomes and early developmental traits. As long as phenotype information is present for individuals in two generations and genotype information is available on the older individual, then it is possible to use this method to estimate both maternal and fetal effects. This could include phenotypes where genome-wide association meta-analyses already exist, such as measures of size at birth including length^12^ and head circumference,^13^ maternal phenotypes during pregnancy such as gestational weight gain,^14^ or developmental phenotypes during childhood such as language development.^15^

The most common study design used when trying to estimate fetal and maternal effects is to have maternal/offspring pairs, with phenotype information on the offspring and genotype information on both the mother and the offspring. These studies are then analysed using a standard linear regression model adjusting for both the maternal and the offspring genotype, which is often referred to as ‘conditional analysis’. One of the benefits of the SEM we describe here is that the coefficients for the maternal and fetal effects are on the same scale as the coefficients from a conditional analysis, and therefore a meta-analysis could be conducted across multiple cohorts with different study designs. Alternatively, because the model can be fit with observed covariance matrices, if the phenotypes of the mother and offspring are both standardized and the effect allele frequency is known, then the summary statistics (allele frequency, beta coefficient from the regression model and variance of the phenotype) from an unconditional analysis for either the fetal effect or the maternal effect can be incorporated into this SEM. This makes it a potentially very powerful approach, as cohorts with phenotype data and genotypes from mother, child or both, can all be incorporated. It also avoids the need to share raw data, which can be problematic for some cohorts, but still allows for all cohorts to be included in the analysis and therefore the sample size maximized.

One of the biggest advantages of this SEM is that it is robust to missing data, either for the individual’s own phenotype or the phenotype of their offspring. This is an advantage over conditional analysis, which uses only those mother/offspring pairs that have genotype data from both persons. It can even be used when no individuals have phenotypes measured on both themselves and their offspring; however, the power to detect a maternal or fetal effect is reduced and a small bias is introduced to the maternal effect estimate. There are unlikely to be many studies with this study design, as the majority would have a combination of individuals with complete data with individuals missing data for their own phenotype or the phenotype of their offspring. Additionally, it is robust to measurement error involving either the individual’s own phenotype or the phenotype of their offspring. However, increasing measurement error in both phenotypes will increase the standard errors and therefore decrease statistical power, similar to the effect of measurement error on ordinary least squares regression.

There are four potential limitations to this SEM. First, although we have shown that the model fits well under a range of minor allele frequencies, in some situations it has difficulty optimizing with low frequency variants (generally with minor allele frequency < 5%). This can often be resolved using the mxTryHard function in OpenMx, which makes several attempts to optimize the model and returns results from the most optimal fit. Second, the SEM assumes multivariate normality between the observed variables and linearity between the genotypes and phenotypes. We have simulated our phenotypes to be normally distributed and ensured that birthweight data in the UK Biobank were approximately normally distributed. In the case of non-normality, an appropriate data transformation can help ensure that the assumption of multivariate normality is satisfied. Third, we assumed additive genetic effects for both the fetal and maternal contributions. An additive model is used in the vast majority of genome-wide association studies in the literature, and theory and data show that the overwhelming majority of genetic loci act in an additive fashion.^16^ If these assumptions do not hold, then we expect reduced power to detect a fetal or maternal effect. Finally, we assume only main effects and no interaction between the maternal and fetal genotypes.

The SEM using observed covariance matrices takes approximately three times longer to compute than an unconditional linear model. Therefore, there is potential for this method to be used in large genomic studies, such as genome-wide studies. A new method for fitting SEMs in genome-wide association studies in a computationally feasible fashion has recently been developed,^17^ which may facilitate analyses involving more complicated models like ours. We note that tests of genetic association have traditionally been performed in the fixed effects part of SEMs (i.e. the ‘model for the means’). In contrast, we have modelled SNP effects in the covariance part of the model, which has allowed us to model latent genotypes. We have shown that within the confines of our study, accuracy of estimates of maternal and fetal effects appear to be robust to the inherent non-normality of individual-level SNP data, which is to be expected in the case of exogenous variables.^18^

A recent study by Horikoshi *et al.*^6^ found three SNPs that were significantly associated with birthweight using the individual’s own genotype (i.e. have a ‘fetal effect’); our analyses indicate that the effects at these loci are driven by a maternal rather than a fetal effect (variants in *MTNR1B*, and near *ACTL9* and *EBF1*). This result is consistent with the conditional analysis of 12 909 mother-offspring pairs in Horikoshi *et al.*^6^ for *MTNR1B* and *EBF1*. The initial finding of a fetal effect appears to be due to the bias in the linear model, and therefore the fetal effect size was approximately half of the maternal effect size estimated using the SEM. We also identified six SNPs where the maternal and fetal effects were in opposite directions (variants in *ADCY5*, *CDKAL1* and *ABCC9* and near *HHEX-IDE*, *ANK1-NKX6-3* and *DTL*). Interestingly, four of these SNPs that exhibited maternal and fetal effects in opposing directions (three of which were confirmed using the conditional analysis in Horikoshi *et al.*^6^) are known type 2 diabetes loci (*ADCY5*, *HHEX*/*IDE, CDKAL1* and *ANK1*), consistent with what is known regarding the underlying biology at these loci.^19^ Importantly, the existence of an opposing maternal effect at these loci would not have been detected had only unconditional linear regressions of offspring birthweight on maternal genotype been performed,^6^ further highlighting the importance of our method in disentangling maternal and fetal effects on perinatal phenotypes.

In summary, we describe a new method for estimating unbiased maternal and fetal effects using studies where genotype data are available for only the individual and not their offspring. We have shown that the type 1 error rate of the method is appropriate, there is substantial statistical power to detect a genetic variant that has a moderate effect on the phenotype and reasonable power to detect whether it is a fetal and/or maternal effect. We have also illustrated that this method could be useful for accurate estimation of fetal and maternal effects in large genetic studies, such as genome-wide association studies, as the computational time is not substantially larger than the standard linear model.

## Funding

N.M.W. is supported by a National Health and Medical Research Council Early Career Fellowship (grant number APP1104818). D.M.E. is funded by an Australian Research Council Future Fellowship (grant number FT130101709) and a Medical Research Council programme grant (grant number MC_UU_12013/4). Access to the UKBB study data was funded by University of Queensland Early Career Researcher Grant (2014002959).

## Supplementary Material

Supplementary Data

## References

[1] Barker DJ, Hales CN, Fall CH, Osmond C, Phipps K, Clark PM. Type 2 (non-insulin-dependent) diabetes mellitus, hypertension and hyperlipidaemia (syndrome X): relation to reduced fetal growth. Diabetologia 1993;36:62–67. 8436255 10.1007/BF00399095

[2] Magnus P. Causes of variation in birth weight: a study of offspring of twins. Clin Genet 1984;25**:** 15–24. 6538464 10.1111/j.1399-0004.1984.tb00457.x

[3] Magnus P. Further evidence for a significant effect of fetal genes on variation in birth weight. Clin Genet 1984;26**:** 289–96. 6541980 10.1111/j.1399-0004.1984.tb01061.x

[4] Lunde A, Melve KK, Gjessing HK, Skjaerven R, Irgens LM. Genetic and environmental influences on birth weight, birth length, head circumference, and gestational age by use of population-based parent-offspring data. Am J Epidemiol 2007;165:734–41. 17311798 10.1093/aje/kwk107

[5] Eaves LJ, Pourcain BS, Davey Smith G, York TP, Evans DM. Resolving the effects of maternal and offspring genotype on dyadic outcomes in genome wide complex trait analysis (‘M-GCTA’). Behav Genet 2014;44:445–55. 25060210 10.1007/s10519-014-9666-6 PMC4174369

[6] Horikoshi M, Beaumont RN, Day FR et al Genome-wide associations for birth weight and correlations with adult disease. Nature 2016;538**:** 248–52. 27680694 10.1038/nature19806 PMC5164934

[7] Allen NE, Sudlow C, Peakman T, Collins R. UK biobank data: come and get it. Sci Transl Med 2014;6**:** 224ed4. 10.1126/scitranslmed.3008601 24553384

[8] Hattersley AT, Beards F, Ballantyne E, Appleton M, Harvey R, Ellard S. Mutations in the glucokinase gene of the fetus result in reduced birth weight. Nat Genet 1998;19**:** 268–70. 9662401 10.1038/953

[9] Pierce BL, VanderWeele TJ. The effect of non-differential measurement error on bias, precision and power in Mendelian randomization studies. Int J Epidemiol 2012;41:1383–93. 23045203 10.1093/ije/dys141

[10] Little RJA, Rubin DB. Statistical Analysis With Missing Data. Hoboken, NJ: Wiley, 1987.

[11] Ounsted M, Scott A, Ounsted C. Transmission through the female line of a mechanism constraining human fetal growth. Ann Hum Biol 1986;13**:** 143–51. 3707043 10.1080/03014468600008281

[12] van der Valk RJ, Kreiner-Moller E, Kooijman MN et al A novel common variant in DCST2 is associated with length in early life and height in adulthood. Hum Mol Genet 2015;24**:** 1155–68. 25281659 10.1093/hmg/ddu510 PMC4447786

[13] Taal HR, St Pourcain B, Thiering E et al Common variants at 12q15 and 12q24 are associated with infant head circumference. Nat Genet 2012;44**:** 532–38. 22504419 10.1038/ng.2238 PMC3773913

[14] Warrington N, Richmond R, Fenstra B et al Maternal and fetal genetic contribution to gestational weight gain. Int J Obes (Lond) 2017, Oct 9. doi: 10.1038/ijo.2017.248. [Epub ahead of print.] 10.1038/ijo.2017.248 PMC5784805 28990592

[15] St Pourcain B, Cents RA, Whitehouse AJ et al *.* Common variation near ROBO2 is associated with expressive vocabulary in infancy. Nat Commun 2014;5**:** 4831. 25226531 10.1038/ncomms5831 PMC4175587

[16] Hill WG, Goddard ME, Visscher PM. Data and theory point to mainly additive genetic variance for complex traits. PLoS Genet 2008;4**:** e1000008. 18454194 10.1371/journal.pgen.1000008 PMC2265475

[17] Verhulst B, Maes HH, Neale MC. GW-SEM: A statistical package to conduct Genome-Wide Structural Equation Modeling. Behav Genet 2017;47**:** 345–59. 28299468 10.1007/s10519-017-9842-6 PMC5423544

[18] Bollen KA. Structural Equations With Latent Variables. Hoboken, NJ: Wiley, 1989.

[19] Beaumont RN, Horikoshi M, McCarthy MI, Freathy RM. How can genetic studies help us to understand links between birth weight and type 2 diabetes? Curr Diabetes Rep 2017;17**:** 22. 10.1007/s11892-017-0852-9 PMC5350261 28293907

