# Supplementary material for: Using structural equation modelling to jointly estimate maternal and fetal effects on birthweight in the UK Biobank
Source: Int J Epidemiol. 2018 Feb 13;47(4):1229–41. doi: 10.1093/ije/dyy015 (PMC6124616; doi:10.1093/ije/dyy015)
Supplement: Supplementary Data [file dyy015_supp.zip › dyy015-suppl_data/ije-2017-07-0762-File008.docx]

**Supplementary Figure 1: Diagram of the structural equation model (SEM) used in the simulation study and the UK Biobank analysis of birthweight when there is missing data.** The SEM in Diagram A) is used to model the subset of individuals with complete data and is the same as Figure 1 in the main manuscript. The SEM in Diagram B) is used to model the subset of genotyped individuals who report their own phenotype, but not their offspring’s phenotype. Genotyped males who report their own phenotype (but not their offspring’s) can be incorporated into this part of the model. The SEM in Diagram C) is used to model the subset of genotyped individuals who report their offspring’s phenotype, but not their own. Phenotype information from male offspring of genotyped mothers can be incorporated into this part of the model. These three models are fit to the three subsets of data that contain the various patterns of missingness, and then the likelihoods from each model are combined. Modelling the data in this way avoids list-wise deletion of cases due to missing phenotype information and makes maximum use of the observed data.

| **A)**  **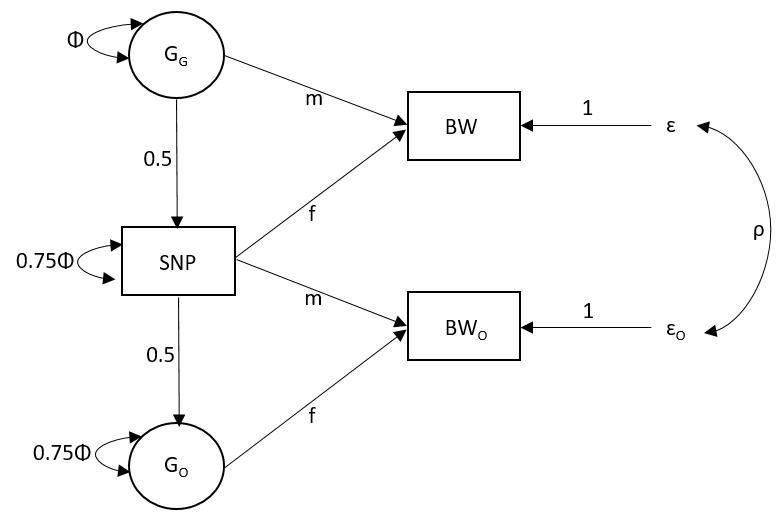** |
| --- |
| **B)**  **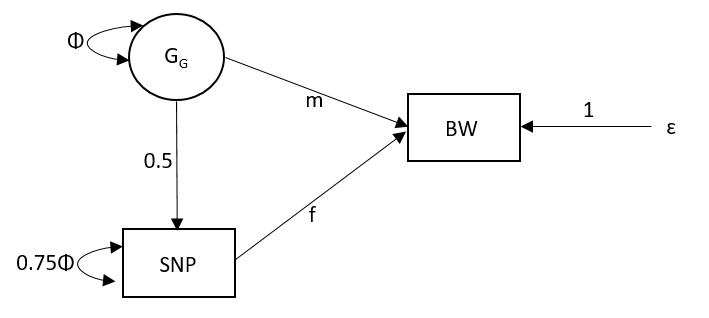** |
| **C)**  **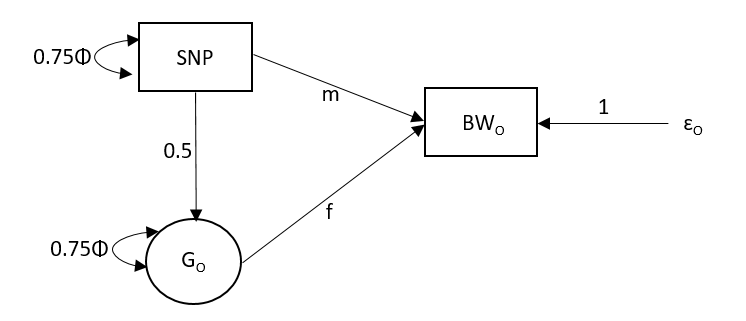** |

**Supplementary Figure 2: Average duration (seconds) from 1,000 simulations to estimate maternal and fetal effects with 95% confidence intervals using an unconditional linear model, the structural equation model (SEM) with covariance matrices and the SEM with raw data. Results are presented for simulations with an allele frequency of either 0.5 or 0.99.**

**
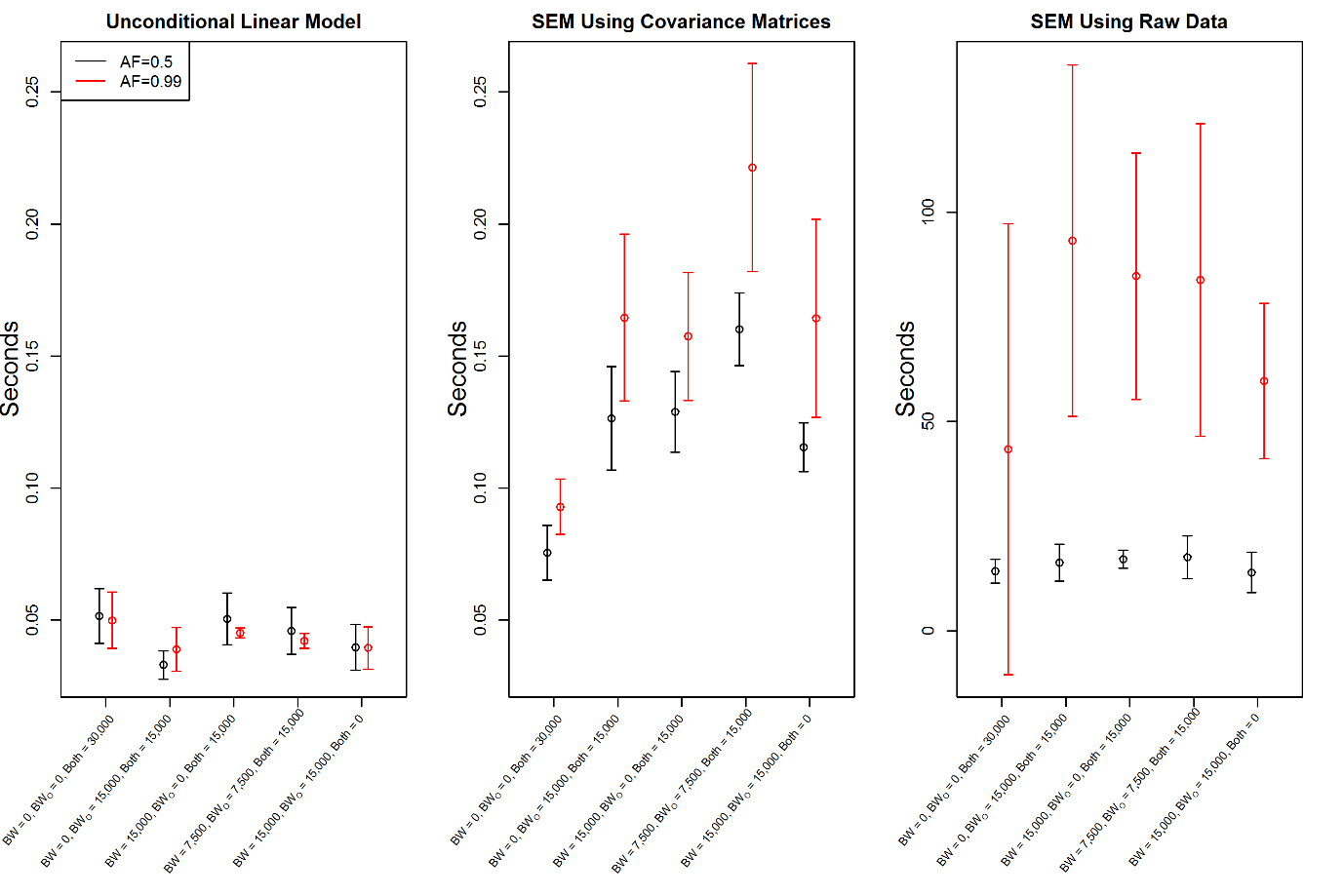
**

**Supplementary Figure 3: Forest plots of the maternal and fetal genetic effects from the linear models and the structural equation model (SEM) for each of the 58 birthweight associated SNPs in the UK Biobank.** Results labelled “Fetal Unadjusted” are those from the linear model assessing the fetal effect; results labelled “Fetal Adjusted” are those from the structural equation model of the fetal effect allowing for maternal effects; results labelled “Maternal Unadjusted” are those from the linear model assessing the maternal effect; results labelled “Maternal Adjusted” are those from the structural equation model of the maternal effect allowing for fetal effects. Loci are classified according to whether they A) exhibit a strong evidence of a maternal effect (some also exhibit a fetal effect), B) are primarily driven through the fetal genotype or C) are difficult to classify. Note the different x-axis values for the *PTCH1*, *YKT6-GCK* and *SUZ12P1-CRLF3* loci.

A)


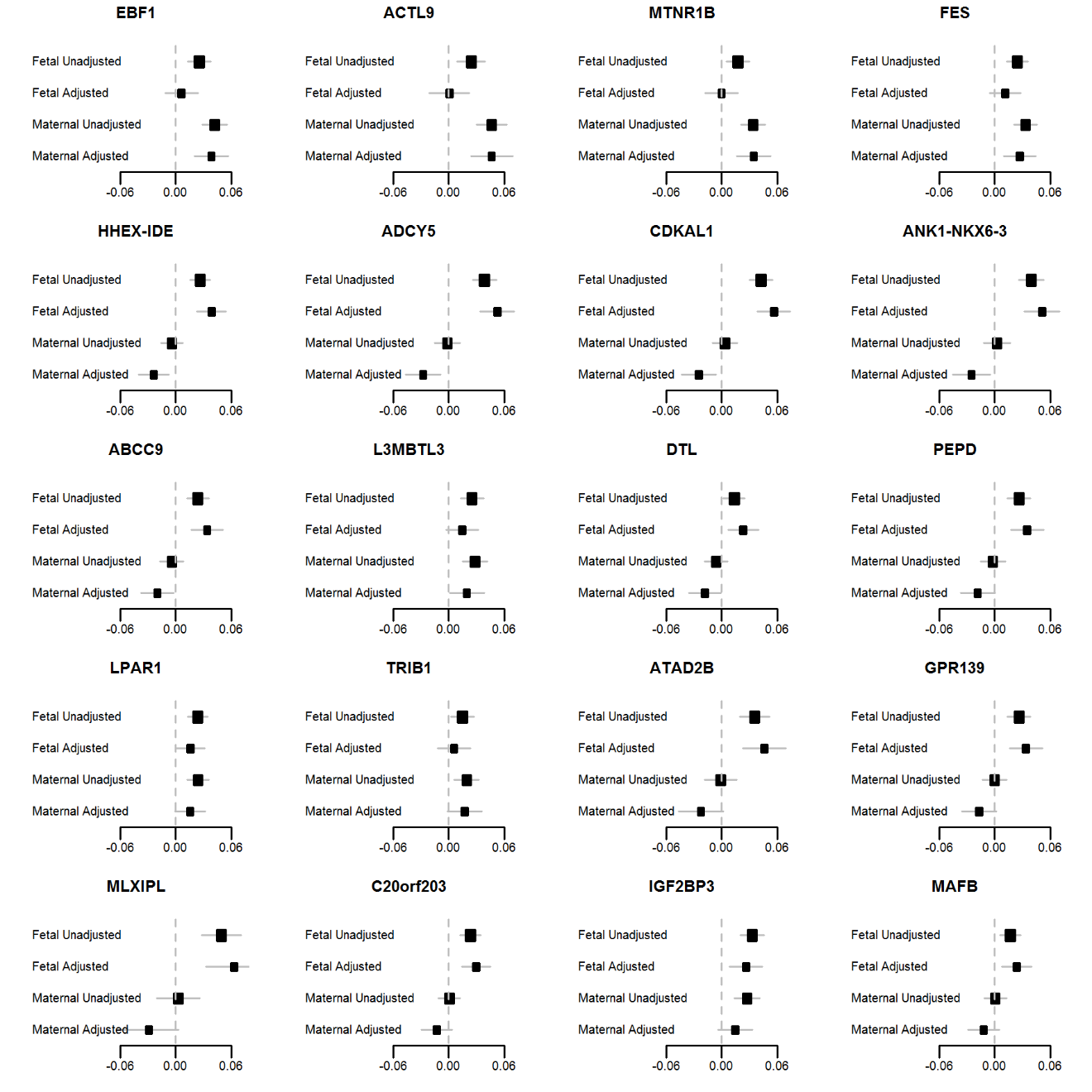


B)


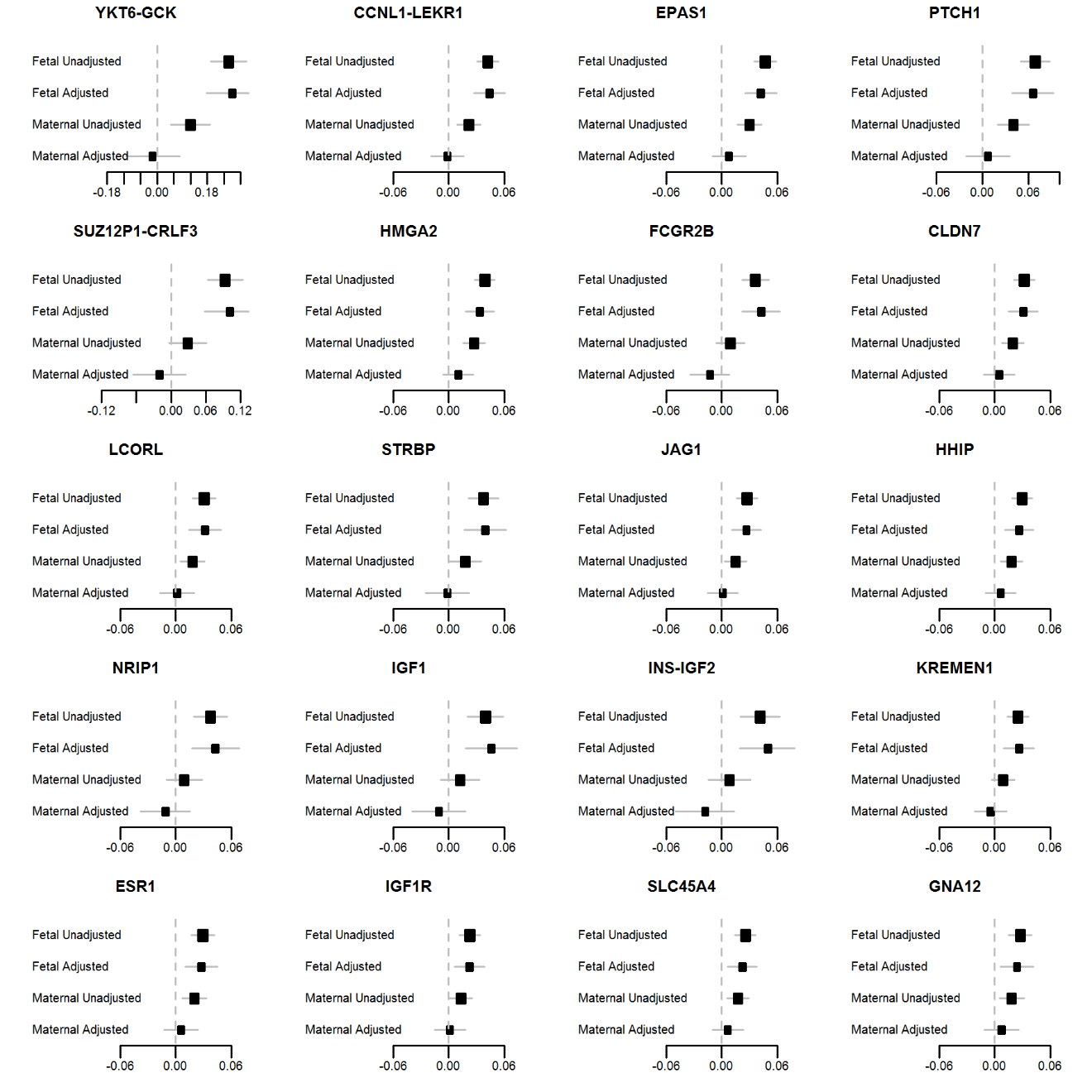


C)


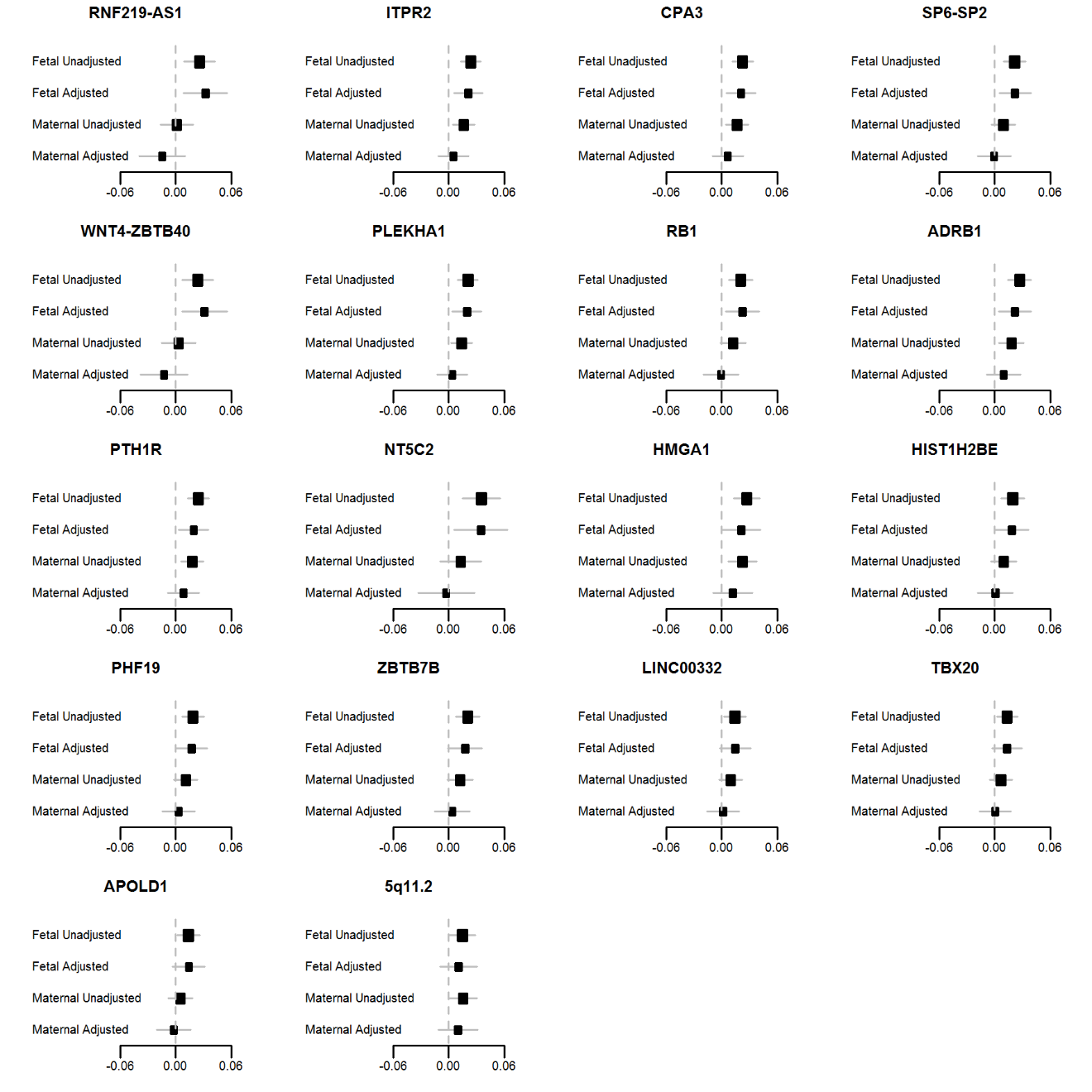


**Supplementary Figure 4: Forest plots of the maternal and fetal genetic effects from the structural equation model for each of the 58 birthweight associated SNPs in the UK Biobank and the conditional linear model in the subset of EGG cohorts with maternal and fetal genotype data (N=12,909 individuals).** Results labelled “Fetal (EGG)” are those obtained from the conditional linear model in EGG that estimates the fetal effect on birthweight adjusting for maternal effects at the same locus (as presented in (1)); results labelled “Fetal (UKBB)” are those obtained by fitting the structural equation model to the UK Biobank data in an attempt to estimate the fetal effect on birthweight; results labelled “Maternal (EGG)” are those from the conditional linear model in EGG that estimates the maternal effect on birthweight adjusting for fetal effects at the same locus (as presented in (1)); results labelled “Maternal (UKBB)” are those obtained by fitting the structural equation model to the UK Biobank data in an attempt to estimate the maternal effect on birthweight. Loci are classified according to whether they A) exhibit strong evidence of a maternal effect (some also exhibit a fetal effect), B) are primarily driven through the fetal genotype and C) are difficult to classify.

A)

**
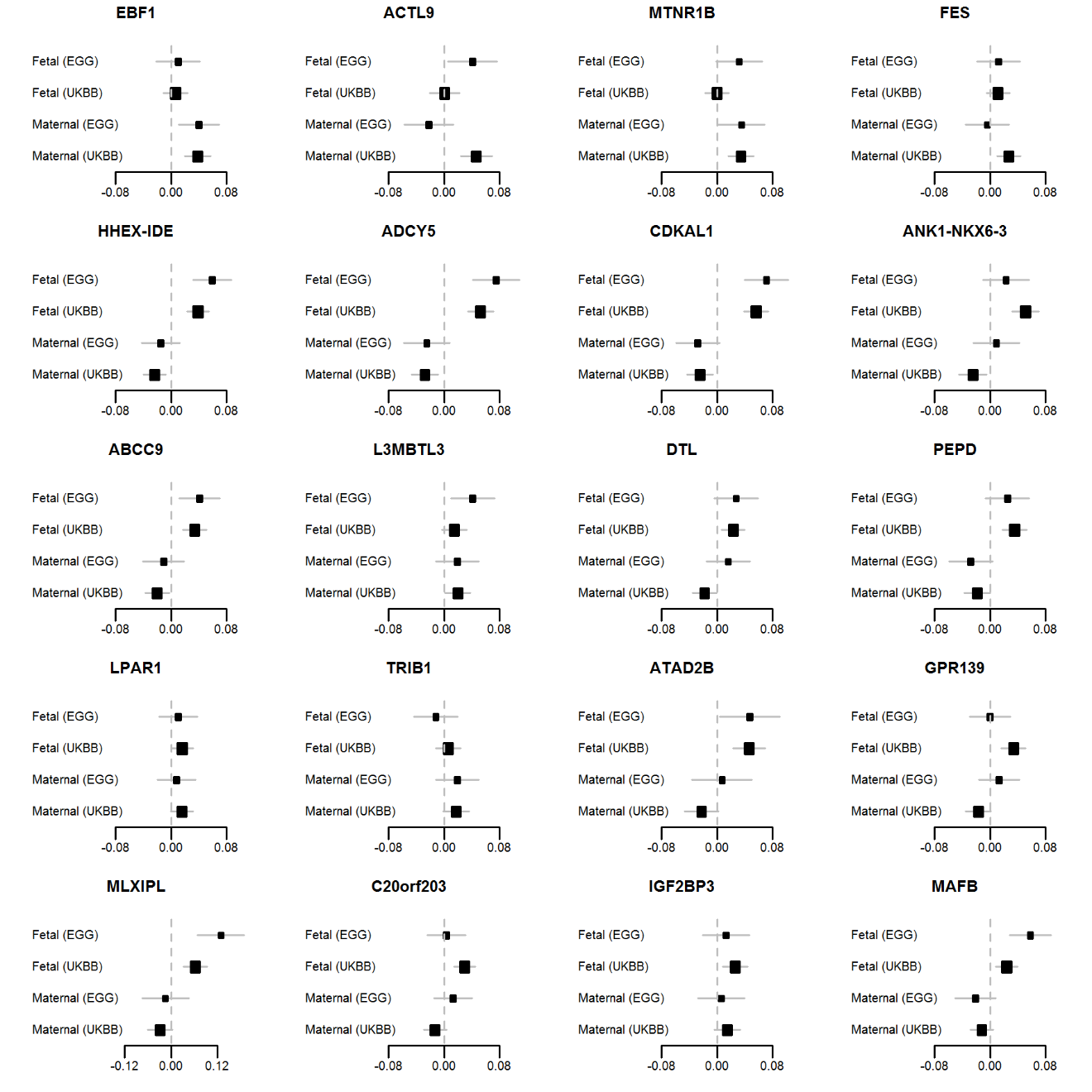
**

B)

**
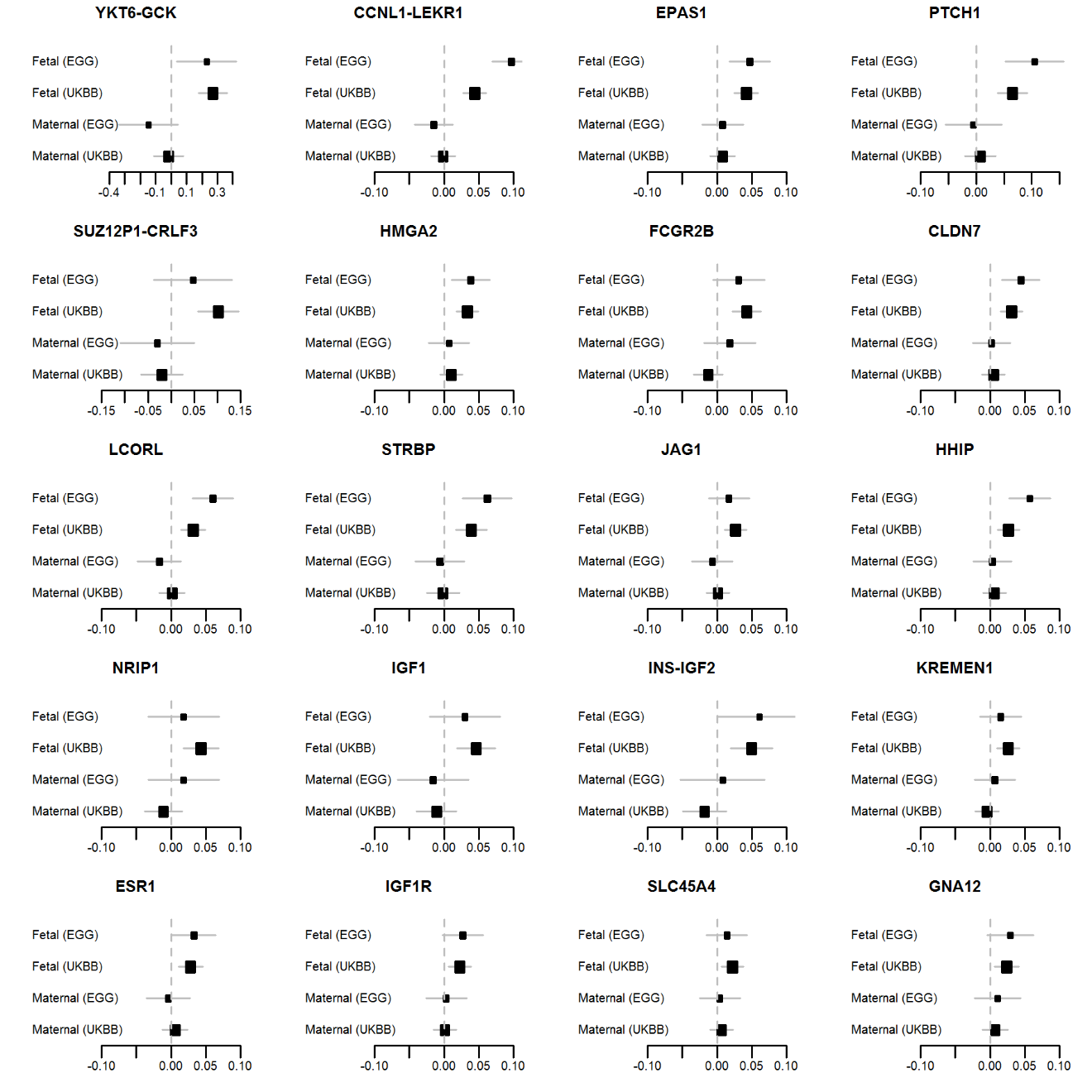
**

C)


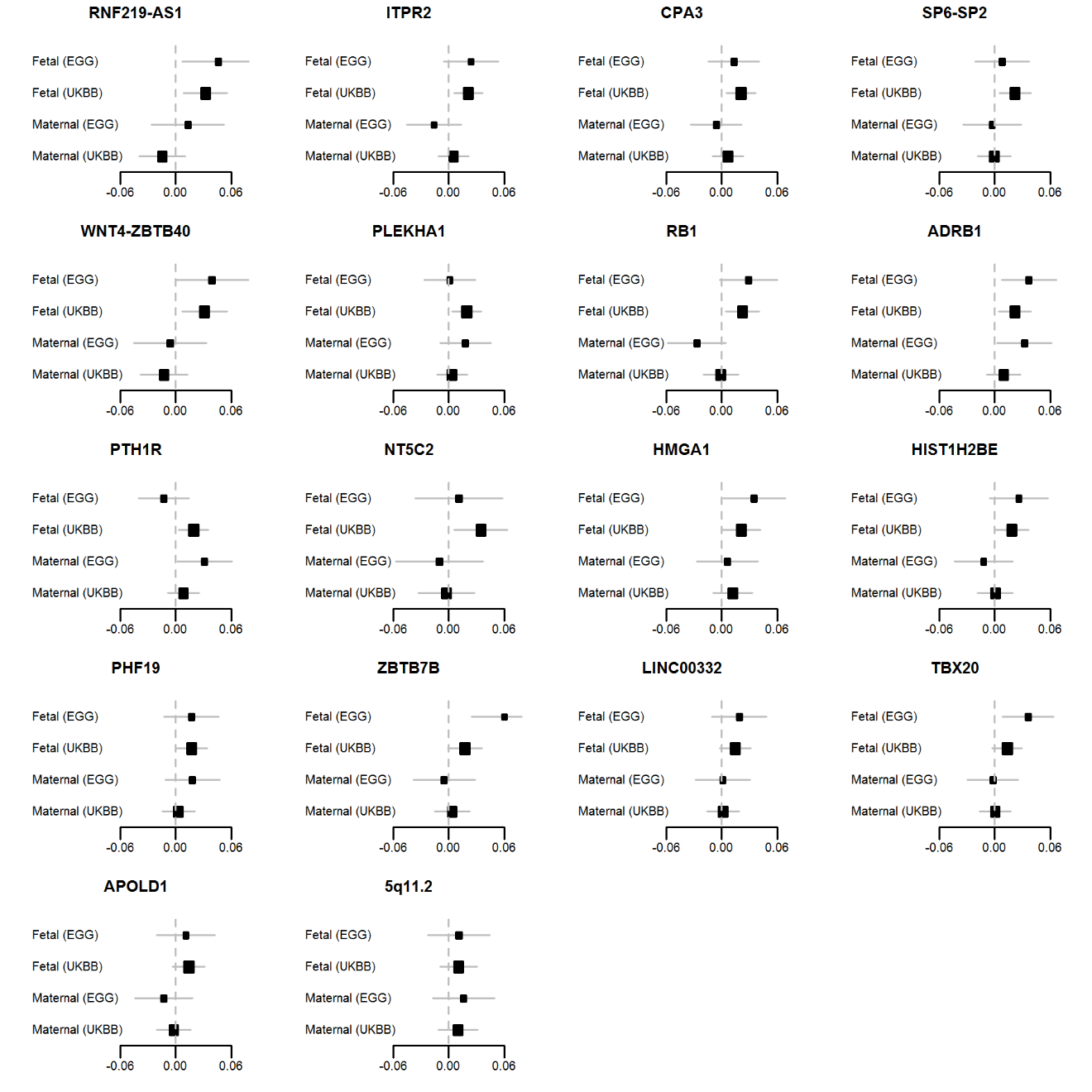


**R code used for the simulation studies:**

library(OpenMx)

*## Simulation code + structural equation model including both maternal and fetal effects using the raw data*

simulate_IV <- function(Nrep = 10000, N = 30000, p=0.5, betazc = 0, betazm = 0, buo = 0.5, bum = 0.5) {

# Model parameters being simulated:

# Nrep = number of repetitions

# N = number of individuals

# p = minor (decreaser) allele freqency

# betazc = child snp effect on 'own' birthweight

# betazm = maternal snp effect on offspring birthweight

# buo = confounding effect of U on offspring birthweight

# bum = confounding effect of U on maternal birthweight

parameter <- data.frame() # data frame for storing the simulated parameters

linmod <- data.frame() # data frame for storing the results from the linear model with maternal SNP on

maternal and offspring BW

base <- data.frame() # data frame for storing the results from the SEM with both maternal and child effects

bzc <- sqrt(abs(betazc)) * sign(betazc) # Path coefficient between SNP and own birthweight ("child effect")

bzm <- sqrt(abs(betazm)) * sign(betazm) # Path coefficient between SNP and offspring birthweight ("maternal

effect")

q <- 1-p # Increaser allele frequency. Assume all SNPs have same variance

a <- sqrt(1/(2*p*q)) # Calculate genotypic value for genetic variable with variance 1

Vem <- (1 - bum^2 - bzc^2 - bzm^2 - bzc*bzm) # Residual variance for maternal birthweight (so variance adds up

to one)

sdem <- sqrt(Vem) # Residual standard error for maternal birthweight

Veo <- (1 - buo^2 - bzc^2 - bzm^2 - bzc*bzm) # Residual variance in offspring birthweight (so variance adds up

to one)

sdeo <- sqrt(Veo) # Residual standard error in offspring birthweight

for(j in 1:Nrep){

### SIMULATE DATA ###

# Simulate grandmother (gm), grandfather (gf) and father (f) SNPs

gm_snp <- sample(x = c(-a,0,a), size = N, replace = TRUE, prob = c(p^2, 2*p*q, q^2))

gf_snp <- sample(x = c(-a,0,a), size = N, replace = TRUE, prob = c(p^2, 2*p*q, q^2))

f_snp <- sample(x = c(-a,0,a), size = N, replace = TRUE, prob = c(p^2, 2*p*q, q^2))

maf_gm <- ((2*(length(which(gm_snp==-a)))) + length(which(gm_snp==0)))/(2*N) # MAF for grandmothers SNP

# Simulate maternal SNP

snp <- vector(length=N)

rm <- runif(N)

for (i in 1:N) {

if((gm_snp[i]==-a) && (gf_snp[i]==-a)) {snp[i] = -a}

if((gm_snp[i]==-a) && (gf_snp[i]==0)) {if(rm[i] <= 0.5) {snp[i] = -a} else {snp[i] = 0}}

if((gm_snp[i]==-a) && (gf_snp[i]==a)) {snp[i] = 0}

if((gm_snp[i]==0) && (gf_snp[i]==-a)) {if(rm[i] <= 0.5) {snp[i] = -a} else {snp[i] = 0}}

if((gm_snp[i]==0) && (gf_snp[i]==0)) {

if(rm[i] <= 0.25) {snp[i] = -a}

if(rm[i] > 0.25 && rm[i] <= 0.75) {snp[i] = 0}

if(rm[i] > 0.75) {snp[i] = a}

}

if((gm_snp[i]==0) && (gf_snp[i]==a)) {if(rm[i] <= 0.5) {snp[i] = a} else {snp[i] = 0}}

if((gm_snp[i]==a) && (gf_snp[i]==-a)) {snp[i] = 0}

if((gm_snp[i]==a) && (gf_snp[i]==0)) {if(rm[i] <= 0.5) {snp[i] = a} else {snp[i] = 0}}

if((gm_snp[i]==a) && (gf_snp[i]==a)) {snp[i] = a}

}

maf_snp <- ((2*(length(which(snp==-a)))) + length(which(snp==0)))/(2*N) # MAF for mothers SNP

# Simulate offspring SNP

go_snp <- vector(length=N)

ro <- runif(N)

for (i in 1:N) {

if((snp[i]==-a) && (f_snp[i]==-a)) {go_snp[i] = -a}

if((snp[i]==-a) && (f_snp[i]==0)) {if(ro[i] <= 0.5) {go_snp[i] = -a} else {go_snp[i] = 0}}

if((snp[i]==-a) && (f_snp[i]==a)) {go_snp[i] = 0}

if((snp[i]==0) && (f_snp[i]==-a)) {if(ro[i] <= 0.5) {go_snp[i] = -a} else {go_snp[i] = 0}}

if((snp[i]==0) && (f_snp[i]==0)) {

if(ro[i] <= 0.25) {go_snp[i] = -a}

if(ro[i] > 0.25 && ro[i] <= 0.75) {go_snp[i] = 0}

if(ro[i] > 0.75) {go_snp[i] = a}

}

if((snp[i]==0) && (f_snp[i]==a)) {if(ro[i] <= 0.5) {go_snp[i] = a} else {go_snp[i] = 0}}

if((snp[i]==a) && (f_snp[i]==-a)) {go_snp[i] = 0}

if((snp[i]==a) && (f_snp[i]==0)) {if(ro[i] <= 0.5) {go_snp[i] = a} else {go_snp[i] = 0}}

if((snp[i]==a) && (f_snp[i]==a)) {go_snp[i] = a}

}

maf_go <- ((2*(length(which(go_snp==-a)))) + length(which(go_snp==0)))/(2*N) # MAF for offspring SNP

U <- rnorm(N, mean=0, sd=1) # Simulate 'confounding' latent variable

bwm <- bzm*gm_snp + bzc*snp + bum*U + rnorm(N, 0, sdem) # Simulate maternal birthweight

bwo <- bzm*snp + bzc*go_snp + buo*U + rnorm(N, 0, sdeo) # Simulate offspring birthweight

### RUN MODELS ###

data_sub <- as.data.frame(cbind(bwm, bwo, snp))

# Linear models for maternal and child effects

lmc <- lm(bwm ~ snp, data_sub)

lmm <- lm(bwo ~ snp, data_sub)

# Set up model parameters

manifests <- names(data_sub)

snps <- names(data_sub)[3]

gg <- paste("gg_", snps, sep="")

go <- paste("go_", snps, sep="")

mvar <- paste("mvar_", snps, sep="")

ovar <- paste("ovar_", snps, sep="")

latents <- c("e1", "e2", go, gg, mvar, ovar)

mean_lab <- paste("mean_", manifests, sep="")

snps_c_bw_lab <- paste("c_", snps, sep="")

snps_m_bw_lab <- paste("m_", snps, sep="")

theta_lab <- paste("theta_", snps, sep="")

# Base model

IVModel_base <- mxModel(model = "IV Model", type="RAM", mxData(observed=raw(data_sub), type="raw"),

mxPath(from=gg, arrows=2, free=TRUE, values=0.2, labels=theta_lab),

#Variance of 'grandmother' latent genetic variables

mxPath(from=mvar, arrows=2, free=TRUE, values=0.2, labels=theta_lab),

#Variance of 'mother' latent genetic variables

mxPath(from=ovar, arrows=2, free=TRUE, values=0.2, labels=theta_lab),

#Variance of 'offspring' latent genetic variables

mxPath(from=mvar, to=snps, arrows=1, free=FALSE, values=sqrt(0.75), labels="MV"),

#Path from maternal latent variable for variance to SNP

mxPath(from=ovar, to=go, arrows=1, free=FALSE, values=sqrt(0.75), labels="OV"),

#Path from offspring latent variable for variance to latent genetic variable

mxPath(from=c("e1", "e2"), arrows=2, free=TRUE, values=1, labels=c("var_e1", "var_e2")),

#Variance of residual errors

mxPath(from="e1", to="e2", arrows=2, free=TRUE, values=0.2, labels=c("phi1")),

#Correlation between residual errors

mxPath(from="e1", to="bwm", arrows=1, free=FALSE, values=1, labels=c("e1")),

#Residual error of maternal birthweight. Value set to 1.

mxPath(from="e2", to="bwo", arrows=1, free=FALSE, values=1, labels=c("e3")),

#Residual error offspring birthweight. Value set to 1.

mxPath(from=snps, to="bwm", arrows=1, free=TRUE, values=0, labels=snps_c_bw_lab),

#Effect of SNPs on maternal BW (child effect)

mxPath(from=snps, to="bwo", arrows=1, free=TRUE, values=0, labels=snps_m_bw_lab),

#Effect of SNPs on offspring BW (maternal effect)

mxPath(from=gg, to="bwm", arrows=1, free=TRUE, values=0, labels=snps_m_bw_lab),

#Effect of grandma genes on maternal BW (maternal effect)

mxPath(from=go, to="bwo", arrows=1, free=TRUE, values=0, labels=snps_c_bw_lab),

#Effect of offspring genes on offspring BW (child effect)

mxPath(from=gg, to=snps, arrows=1, free=FALSE, values=0.5, labels="G"),

#Path from grandma genes to maternal SNPs. Value set to 0.5

mxPath(from=snps, to=go, arrows=1, free=FALSE, values=0.5, labels="O"),

#Path from maternal SNPs to offspring genes. Value set to 0.5

mxPath( from="one", to=manifests, arrows=1, free=TRUE, values=rep(0,length(manifests)), labels=mean_lab),

# means and intercepts

manifestVars = manifests, latentVars = latents

)

IVFit_base <- mxRun(IVModel_base)

Zscore_base <- summary(IVFit_base)$parameters[,5]/summary(IVFit_base)$parameters[,6]

Pval_base <- 2*(1-pnorm(abs(Zscore_base),0,1))

# Save results

parameter <- rbind(parameter, c(j, maf_gm, maf_snp, maf_go, cor(snp, gm_snp), cor(go_snp, snp),

cor(gm_snp,go_snp), mean(bwm), sd(bwm), mean(bwo), sd(bwo), cor(bwm, bwo)))

linmod <- rbind(linmod, c(summary(lmc)$coefficients[2,1], summary(lmc)$coefficients[2,2],

summary(lmc)$coefficients[2,4], summary(lmm)$coefficients[2,1], summary(lmm)$coefficients[2,2], summary(lmm)$coefficients[2,4]))

base <- rbind(base, c(summary(IVFit_base)$parameters[1,5], summary(IVFit_base)$parameters[1,6],

Pval_base[1], summary(IVFit_base)$parameters[2,5], summary(IVFit_base)$parameters[2,6],

Pval_base[2]))

}

names(parameter) <- c("Repeat_Number", "MAF_GM", "MAF_Mat", "MAF_Off", "Cor_GM_Mat", "Cor_Mat_Off", "Cor_GM_Off",

"Mean_BWM", "SD_BWM", "Mean_BWO", "SD_BWO", "Cor_BWM_BWO")

names(linmod) <- c("Beta_lm_c", "SE_lm_c", "P_lm_c", "Beta_lm_m", "SE_lm_m", "P_lm_m")

names(base) <- c("Beta_base_c", "SE_base_c", "P_wald_base_c","Beta_base_m", "SE_base_m", "P_wald_base_m")

out=cbind(parameter, linmod, base)

out.fname = paste("Simulations_N_", N, "_MAF_", p,"_Confound_", buo, "_BetaZC_", betazc, "_BetaZM_", betazm,

sep="")

outfile = paste(out.fname,"txt", sep=".")

write.table(out,file=outfile, row.names=F, sep="\t")

}

*## Structural equation model including both maternal and fetal effects using covariance matrices for each subset of complete data*

# Data is simulated the same as above, however data_sub is now split into three subsets of data:

# data_sub1 = dataset of individuals with both mother and offspring birthweight; all three columns of data available

# data_sub2 = dataset of individuals with only maternal birthweight; only bwm and snp columns available

# data_sub3 = dataset of individuals with only offspring birthweight; only bwo and snp columns available

# Complete data (i.e., data on both mums and offspring)

snps <- names(data_sub)[3]

gg <- paste("gg_", snps, sep="")

go <- paste("go_", snps, sep="")

mvar <- paste("mvar_", snps, sep="")

ovar <- paste("ovar_", snps, sep="")

snps_c_bw_lab <- paste("c_", snps, sep="")

snps_m_bw_lab <- paste("m_", snps, sep="")

theta_lab <- paste("theta_", snps, sep="")

manifests_complete <- names(data_sub1)

latents_complete <- c("e1", "e2", go, gg, mvar, ovar)

mean_lab_complete <- paste("mean_", manifests_complete, sep="")

IVModel_complete <- mxModel(model = "complete", type="RAM", mxData(observed=cov(data_sub1), type="cov",

numObs=nrow(data_sub1), means=colMeans(data_sub1)),

mxPath(from=gg, arrows=2, free=TRUE, values=0.2, labels=theta_lab),

#Variance of 'grandmother' latent genetic variables

mxPath(from=mvar, arrows=2, free=TRUE, values=0.2, labels=theta_lab),

#Variance of 'mother' latent genetic variables

mxPath(from=ovar, arrows=2, free=TRUE, values=0.2, labels=theta_lab),

#Variance of 'offspring' latent genetic variables

mxPath(from=mvar, to=snps, arrows=1, free=FALSE, values=sqrt(0.75), labels="MV"),

#Path from maternal latent variable for variance to SNP

mxPath(from=ovar, to=go, arrows=1, free=FALSE, values=sqrt(0.75), labels="OV"),

#Path from offspring latent variable for variance to latent genetic variable

mxPath(from=c("e1", "e2"), arrows=2, free=TRUE, values=1, labels=c("var_e1", "var_e2")),

#Variance of residual errors

mxPath(from="e1", to="e2", arrows=2, free=TRUE, values=0.2, labels=c("phi1")),

#Correlation between residual errors

mxPath(from="e1", to="bwm", arrows=1, free=FALSE, values=1, labels=c("e1")),

#Residual error of maternal birthweight. Value set to 1.

mxPath(from="e2", to="bwo", arrows=1, free=FALSE, values=1, labels=c("e3")),

#Residual error offspring birthweight. Value set to 1.

mxPath(from=snps, to="bwm", arrows=1, free=TRUE, values=0, labels=snps_c_bw_lab),

#Effect of SNPs on maternal BW (child effect)

mxPath(from=snps, to="bwo", arrows=1, free=TRUE, values=0, labels=snps_m_bw_lab),

#Effect of SNPs on offspring BW (maternal effect)

mxPath(from=gg, to="bwm", arrows=1, free=TRUE, values=0, labels=snps_m_bw_lab),

#Effect of grandma genes on maternal BW (maternal effect)

mxPath(from=go, to="bwo", arrows=1, free=TRUE, values=0, labels=snps_c_bw_lab),

#Effect of offspring genes on offspring BW (child effect)

mxPath(from=gg, to=snps, arrows=1, free=FALSE, values=0.5, labels="G"),

#Path from grandma genes to maternal SNPs. Value set to 0.5

mxPath(from=snps, to=go, arrows=1, free=FALSE, values=0.5, labels="O"),

#Path from maternal SNPs to offspring genes. Value set to 0.5

mxPath( from="one", to=manifests_complete, arrows=1, free=TRUE, values=rep(0,length(manifests_complete)),

labels=mean_lab_complete), # means and intercepts

manifestVars = manifests_complete, latentVars = latents_complete

)

# Data on Mums only

manifests_mums <- names(data_sub2)

latents_mums <- c("e1", gg, mvar)

mean_lab_mums <- paste("mean_", manifests_mums, sep="")

IVModel_mums <- mxModel(model = "mums", type="RAM", mxData(observed=cov(data_sub2), type="cov",

numObs=nrow(data_sub2), means=colMeans(data_sub2)),

mxPath(from=gg, arrows=2, free=TRUE, values=0.2, labels=theta_lab),

#Variance of 'grandmother' latent genetic variables

mxPath(from=mvar, arrows=2, free=TRUE, values=0.2, labels=theta_lab),

#Variance of 'mother' latent genetic variables

mxPath(from=mvar, to=snps, arrows=1, free=FALSE, values=sqrt(0.75), labels="MV"),

#Path from maternal latent variable for variance to SNP

mxPath(from="e1", arrows=2, free=TRUE, values=1, labels="var_e1"), #Variance of residual errors

mxPath(from="e1", to="bwm", arrows=1, free=FALSE, values=1, labels="e1"),

#Residual error of maternal birthweight. Value set to 1.

mxPath(from=snps, to="bwm", arrows=1, free=TRUE, values=0, labels=snps_c_bw_lab),

#Effect of SNPs on maternal BW (child effect)

mxPath(from=gg, to="bwm", arrows=1, free=TRUE, values=0, labels=snps_m_bw_lab),

#Effect of grandma genes on maternal BW (maternal effect)

mxPath(from=gg, to=snps, arrows=1, free=FALSE, values=0.5, labels="G"),

#Path from grandma genes to maternal SNPs. Value set to 0.5

mxPath( from="one", to=manifests_mums, arrows=1, free=TRUE, values=rep(0,length(manifests_mums)),

labels=mean_lab_mums), # means and intercepts

manifestVars = manifests_mums, latentVars = latents_mums

)

# Data on Offspring only

manifests_off <- names(data_sub3)

latents_off <- c("e2", go, ovar)

mean_lab_off <- paste("mean_", manifests_off, sep="")

IVModel_offspring <- mxModel(model = "offspring", type="RAM", mxData(observed=cov(data_sub3), type="cov",

numObs=nrow(data_sub3), means=colMeans(data_sub3)),

mxPath(from=snps, arrows=2, free=TRUE, values=0.2, labels=theta_lab),

#Variance of 'mother' genetic variables

mxPath(from=ovar, arrows=2, free=TRUE, values=0.2, labels=theta_lab),

#Variance of 'offspring' latent genetic variables

mxPath(from=ovar, to=go, arrows=1, free=FALSE, values=sqrt(0.75), labels="OV"),

#Path from offspring latent variable for variance to latent genetic variable

mxPath(from="e2", arrows=2, free=TRUE, values=1, labels="var_e2"), #Variance of residual errors

mxPath(from="e2", to="bwo", arrows=1, free=FALSE, values=1, labels=c("e3")),

#Residual error offspring birthweight. Value set to 1.

mxPath(from=snps, to="bwo", arrows=1, free=TRUE, values=0, labels=snps_m_bw_lab),

#Effect of SNPs on offspring BW (maternal effect)

mxPath(from=go, to="bwo", arrows=1, free=TRUE, values=0, labels=snps_c_bw_lab),

#Effect of offspring genes on offspring BW (child effect)

mxPath(from=snps, to=go, arrows=1, free=FALSE, values=0.5, labels="O"),

#Path from maternal SNPs to offspring genes. Value set to 0.5

mxPath( from="one", to=manifests_off, arrows=1, free=TRUE, values=rep(0,length(manifests_off)),

labels=mean_lab_off), # means and intercepts

manifestVars = manifests_off, latentVars = latents_off

)

# Combine groups

minus2ll <- mxAlgebra(expression=complete.fitfunction + mums.fitfunction + offspring.fitfunction,

name="minus2loglikelihood" )

obj <- mxFitFunctionAlgebra("minus2loglikelihood")

model <- mxModel(model="IV Model", IVModel_complete, IVModel_mums, IVModel_offspring, minus2ll, obj)

# Run model

IVFit_base <- mxRun(model)

Zscore_base <- summary(IVFit_base)$parameters[,5]/summary(IVFit_base)$parameters[,6]

Pval_base <- 2*(1-pnorm(abs(Zscore_base),0,1))

**References:**

1. Horikoshi M, Beaumont RN, Day FR, Warrington NM, Kooijman MN, Fernandez-Tajes J, et al. Genome-wide associations for birth weight and correlations with adult disease. Nature. 2016;538(7624):248-52.
